# Transcriptional Profiling Suggests Extensive Metabolic Rewiring of Human and Mouse Macrophages during Early Interferon Alpha Responses

**DOI:** 10.1155/2018/5906819

**Published:** 2018-07-25

**Authors:** Duale Ahmed, Allison Jaworski, David Roy, William Willmore, Ashkan Golshani, Edana Cassol

**Affiliations:** ^1^Department of Biology, Carleton University, Ottawa, ON, Canada; ^2^Department of Health Sciences, Carleton University, Ottawa, ON, Canada; ^3^Institute of Biochemistry, Carleton University, Ottawa, ON, Canada

## Abstract

Emerging evidence suggests that cellular metabolism plays a critical role in regulating immune activation. Alterations in energy and lipid and amino acid metabolism have been shown to contribute to type I interferon (IFN) responses in macrophages, but the relationship between metabolic reprogramming and the establishment of early antiviral function remains poorly defined. Here, we used transcriptional profiling datasets to develop global metabolic signatures associated with early IFN-*α* responses in two primary macrophage model systems: mouse bone marrow-derived macrophages (BMM) and human monocyte-derived macrophages (MDM). Short-term stimulation with IFN-*α* (<4 hours) was associated with significant metabolic rewiring, with >500 metabolic genes altered in mouse and human macrophage models. Pathway and network analysis identified alterations in genes associated with cellular bioenergetics, cellular oxidant status, cAMP/AMP and cGMP/GMP ratios, branched chain amino acid catabolism, cell membrane composition, fatty acid synthesis, and *β*-oxidation as key features of early IFN-*α* responses. These changes may have important implications for initial establishment of antiviral function in these cells.

## 1. Introduction

Type I interferons (IFN) (IFN-*α* and IFN-*β*) play a seminal role in antiviral, antibacterial, and antitumour responses and act as critical regulators of the innate and adaptive immune system [1–5]. These pleiotropic cytokines are produced following engagement of pattern recognition receptors and signal through the ubiquitously expressed transmembrane IFN-*α* receptor (IFNAR), composed of IFNAR1 and IFNAR2 subunits [5–7]. Cellular responses to type I IFN are cell type- and context-dependent and vary during the course of an immune response [8–11]. The variability in these responses are due, in part, to the cumulative effects of JAK-STAT, the p38 MAP kinase (MAPK), the MAP kinase kinase/ERK/MAPK signal-interacting kinase, and the phosphatidylinositol 3-kinase (PI3K)/AKT/mammalian target of rapamycin (mTOR) signaling pathways [11–13].

Emerging evidence suggests that cellular metabolism plays a critical role in regulating and fine tuning immune function [14–16]. Alterations in cellular bioenergetics, amino acid metabolism, and lipid metabolism have been shown to affect cytokine production, signaling protein activity, and cell differentiation [17–19]. In macrophages, stimulation with type I IFNs has been shown to increase glycolytic flux, inhibit sterol biosynthesis, shift lipid metabolism from de novo synthesis to lipid import, and increase tryptophan catabolism [20–25]. This metabolic reprogramming is required to mount functional antiviral responses and has been shown to regulate antigen presentation, inflammatory mediator production, phagocytosis efficiency, and intracellular killing [26, 27].

Recent studies suggest metabolic adaptations in macrophages occur at the molecular level (i.e., gene expression) very early during the process activation and functional polarization [28–30]. In lipopolysaccharide- (LPS-) stimulated macrophages, cellular activation has been shown to undergo stages of time-resolved metabolic reprogramming into initiation, early, and amplification phases [28]. To date, there has been no systematic characterization of metabolic reprogramming associated with type I IFN responses, particularly when examined as a function of time. In the current study, we used transcriptional profiling to evaluate global changes in metabolic gene expression following short-term IFN-*α* stimulation (<4 hours) in two well-characterized primary macrophage model systems: mouse bone marrow-derived macrophages (BMM) and human monocyte-derived macrophages (MDM). Our findings provide a systematic understanding of altered metabolic genes associated with early IFN-*α* responses in BMM and MDM and identify potential metabolic mechanisms that may contribute to initial establishment of antiviral immune responses.

## 2. Materials and Methods

### 2.1. Microarray Normalization and Processing

Microarray datasets were extracted from the Gene Expression Omnibus (GEO) repository from the National Center for Biotechnology Information (NCBI). Datasets were identified using the search terms “Macrophage Interferon” and “Type I Interferon” [31]. A total of 10 datasets were identified using these search criteria. Two studies were identified that assessed short-term stimulation (<4 hours) in either mouse bone marrow-derived macrophages (BMM) or human monocyte-derived macrophages (MDM). The selected BMM dataset [32] differentiated bone marrow cells in DMEM media with 10 ng/mL of macrophage colony-stimulating factor (M-CSF), 10% FBS, and 1% penicillin/streptomycin for seven days before replacing the media on day six. BMM were stimulated with 62 U/mL IFN-*α* (approximately 1 ng/mL) for 2.5 hours prior to cell lysis and RNA extraction (Supplementary Figure S1). The selected MDM dataset [33] isolated monocytes *via* adherence (4 hours) and differentiated cells in DMEM media with 2 mM L-glutamine, 100 U/mL penicillin, 100 *μ*g/mL streptomycin, and 10% FCS for 7 days. After differentiation, MDM were treated with 10 ng/mL IFN-*α* for 4 hours prior to cell lysis and RNA extraction (Supplementary Figure S1). Raw gene expression data was normalized by median centering, and preprocessing was performed in dChip [34]. Probes were excluded from the analysis when present calls (*p* < 0.05) were identified in less than 20% of samples.

### 2.2. Identification of Significant Metabolic Genes in IFN-Stimulated Macrophages

**Figure 1:**
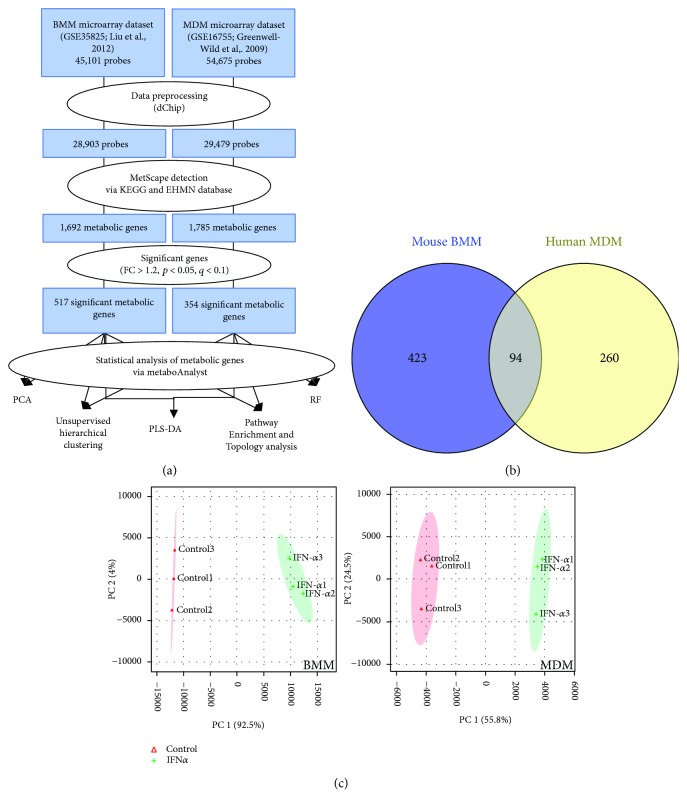
Short term IFN-*α* stimulation is associated with altered expression of metabolic genes in human monocyte-derived macrophages (MDM) and mouse bone marrow-derived macrophages (BMM). (a) Workflow used to identify differentially expressed metabolic genes in IFN-*α*-stimulated mouse BMM (2.5 hours) and human MDM (4 hours). Metabolic genes were identified in MetScape using the Kyoto Encyclopedia of Genes and Genomes (KEGG) and the Edinburgh Human Metabolic Network (EHMN) databases. (b) Venn diagram showing the number of metabolic genes common to the BMM (yellow) and MDM (blue) datasets (FC > 1.2, *p* < 0.05, FDR < 0.10). (c) Principal component analysis (PCA) of metabolic gene sets from BMM (left) and MDM (right) following IFN-*α* stimulation (*n* = 517 and 354 metabolic genes in the BMM and MDM datasets, resp.).

Metabolic genes were identified using the MetScape plugin in Cytoscape [35–37]. These genes were identified by mapping Entrez Gene IDs to metabolic pathways found in the Kyoto Encyclopedia of Genes and Genomes (KEGG) and Edinburgh Human Metabolic Network (EHMN) databases. Manual curation was performed to identify metabolic genes that did not map to KEGG or EHMN pathways. Fold change (FC) analyses, *p* values, and false discovery rates (FDR) were calculated in R. Differentially expressed genes were defined as a −1.2 ≤ FC ≥1.2, *p* value ≤ 0.05, and FDR ≤ 0.1. This fold change cutoff was selected to be inclusive of small differences in gene expression. Biologically relevant alterations in metabolic gene expression were identified by combining FC with pathway and network analyses. Classification analyses including principal component analysis (PCA), partial least squares-discriminant analysis (PLS-DA), random forest (RF), and unsupervised hierarchical clustering were performed in MetaboAnalyst [38]. Gene Set Enrichment Analysis (GSEA) [39] was performed using the hallmark gene sets (H; *n* = 50), the curated gene sets (C2; *n* = 4731), and the GO gene sets (C5; *n* = 5917) from Molecular Signatures Database (MSigDB) version 6.0. Significant enrichment was defined as a *p* ≤ 0.05 and FDR < 0.25. Metabolic pathway enrichment and topology analysis were performed in MetaboAnalyst. Significant enrichment of metabolic datasets was defined as *p* ≤ 0.05. Metabolic network annotation and analysis were performed in Cytoscape and the Database for Annotation, Visualization and Integrated Discovery (DAVID) [40, 41]. The work flow is shown in Figure 1(a).

## 3. Results

### 3.1. Type I IFN Stimulation of Mouse BMM and Human MDM Is Associated with Enrichment of Metabolic Gene Sets

Of the >45,000 unique probe sets analyzed across the two datasets, 28,903 and 29,479 probes were identified as present in the BMM and MDM datasets, respectively. In total, 7338 genes were differentially expressed in IFN-*α*-stimulated BMM compared to unstimulated controls (Supplementary Table S1). Conversely, 3804 genes were differentially expressed in IFN-*α* stimulated MDM compared to controls (Supplementary Table S2). GSEA identified significantly enriched gene sets within each dataset. Two hundred and eighty-five gene sets were enriched in BMM distributed across three main functional categories including immune signaling and function (*n* = 151), cellular metabolism (*n* = 40), and other biological states and processes (*n* = 94). Enriched metabolic processes in BMM included glycolysis and gluconeogenesis, the regulation of nitric oxide biosynthetic process, and tryptophan, arginine, and proline metabolism (Supplementary Table S3). In MDM, GSEA identified 948 enriched gene sets (immune signaling and function: *n* = 360, cellular metabolism: *n* = 107, and other biological states and processes: *n* = 481). Enriched metabolic processes included reactive oxygen species (ROS) metabolism and biosynthesis, tryptophan metabolism, regulation of steroid biosynthetic process, and regulation of oxidoreductase activity (Supplementary Table S4).

### 3.2. Metabolic Genes Represent Important Classifiers of Early Type I IFN Responses in BMM and MDM

**Figure 2:**
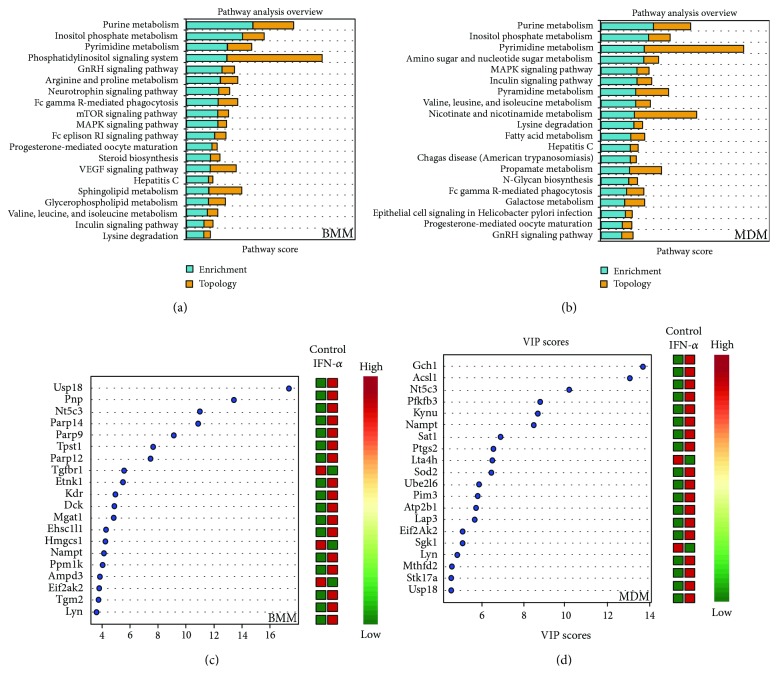
Metabolic genes are top classifiers of IFN-*α* stimulation in BMM and MDM. (a) Pathway enrichment and topology analysis of mouse BMM and human MDM following IFN-*α* stimulation (*p* < 0.05). Analyses were performed using all metabolic genes. The blue bars represent enrichment analysis. The yellow bars represent topology scores. (b) Top metabolic classifiers of IFN-*α* stimulation were identified using variable importance in projection (VIP) scores based on PLS-DA models (*p* < 0.05). Analyses were performed using all metabolic genes (*p* < 0.05). Red and green in the heat map represent upregulation and downregulation of gene expression, respectively.

Given the significant enrichment of metabolic gene sets in GSEA analysis, we used MetScape to identify all metabolic genes detected across datasets. Of the >1600 metabolic genes identified, 517 and 354 were altered following short-term IFN-*α* stimulation of BMM and MDM, respectively (−1.2 ≤ FC ≥ 1.2, *p* value ≤ 0.05, FDR ≤ 0.1). Ninety-four genes were altered in both datasets with the same directionality (either upregulated or downregulated) (Figure 1(b)) including genes associated with cellular bioenergetics (*PFKFB3*, *PDP*), tryptophan metabolism (*KMO*, *WARS*), nucleotide metabolism (*NT5C3*, *CNP*), and lipid metabolism (*SPTLC2*, *AGPAT5*, *SQLE*, and *SOAT1*). PCA showed a clear separation between the control and IFN-*α*-treated samples in both BMM and MDM based on the metabolic gene subset (Figure 1(c)). Similarly, random forest analysis classified control and IFN-*α*-stimulated cells with 100% predictive accuracy using metabolic genes. Variable importance in projection (VIP) analysis identified genes involved in nucleotide degradation (*PNP*, *AMPD3*) and lipid metabolism (*ETNK1*, *HMGCS1*) as top classifiers in IFN-*α* stimulation in BMM. Top classifiers in MDM included genes associated with glycolysis (*PFKFB3*), tryptophan catabolism (*KYNU*), and reactive oxygen species production (*GCH1*, *SOD2*) (Figures 2(c) and 2(d)). Only *EIF2AK2*, *NAMPT*, and *NT5C3* and the ubiquitin-related gene *USP18* overlapped as top classifiers across datasets. EIF2AK2 is involved in mRNA translation and inflammasome activation [42], NAMPT is a key NAD^+^producing gene [43], and NT5C3 is an antiviral pyrimidine nucleotidase [44].

Pathway enrichment and topology analysis identified enrichment in purine, pyrimidine, inositol phosphate, and branched-chain amino acid metabolism in addition to lysine degradation in both BMM and MDM. Metabolic pathways uniquely enriched in BMM included arginine and proline metabolism, steroid biosynthesis, sphingolipid and glycerophospholipid metabolism (Figure 2(a), Supplementary Figure S2). Metabolic pathways uniquely enriched in MDM included amino sugar and nucleotide sugar metabolism, nicotinate and nicotinamide metabolism, galactose metabolism, and fatty acid (FA) metabolism (Figure 2(b), Supplementary Figure S2).

### 3.3. Short Term IFN-*α* Stimulation Alters Genes Associated with Energy Metabolism in BMM and MDM

**Figure 3:**
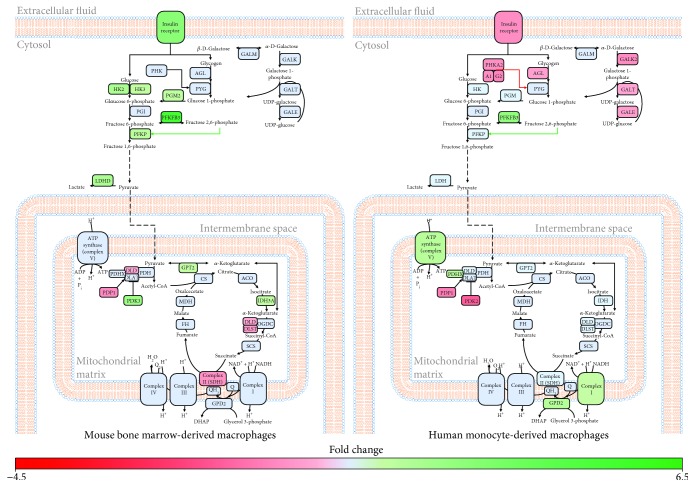
Genes associated with bioenergetic processes are differentially expressed in mouse BMM and human MDM following IFN-*α* stimulation. Significantly altered (−1.2 ≤ FC ≥ 1.2, *p* value ≤ 0.05, FDR ≤ 0.1) metabolic genes involved in energy production were mapped to their respective pathways using MetScape and DAVID. Green and red represent genes that have been significantly upregulated or downregulated, respectively. Blue represents genes that were not altered following IFN-*α* stimulation.

To better functionally characterize differential gene expression in IFN-*α*-stimulated BMM and MDM, altered genes (−1.2 ≤ FC ≥ 1.2, *p* value ≤ 0.05, FDR ≤ 0.1) were mapped to metabolic pathways and networks. Consistent with the literature [20], short-term IFN-*α* stimulation of BMM was associated with an upregulation of glycolytic genes (*HK2*, *HK3*, *PGM2*, *PFKP*, *PFKFB3*, and *INSR*) compared to unstimulated controls (Figure 3, Supplementary Figure S3). Key genes involved in pyruvate metabolism were also altered in BMM following IFN-*α* stimulation. Whereas pyruvate dehydrogenase kinase 3 (*PDK3*) was upregulated, pyruvate dehydrogenase phosphatase 1 (*PDP1*) and dihydrolipoamide dehydrogenase (*DLD*) were downregulated. These alterations may affect the activity of the pyruvate dehydrogenase complex (PDH) and increase lactate production. Consistent with these findings, lactate dehydrogenase D (*LDHD*) was also upregulated in IFN-*α*-stimulated BMM. Interestingly, IFN-*α*-stimulated BMMs also upregulated levels of isocitrate dehydrogenase (*IDH3A*) and the downregulation of *DLD* and dihydrolipoamide *S*-succinyltransferase (*DLST*) expression. DLD and DLST are key components of the oxoglutarate dehydrogenase complex (OGDC) and play an important role in converting 2-oxoglutarate to succinyl-CoA. Along the succinate-fumarate-malate axis, succinate dehydrogenase complex subunit A (*SDHA*) was downregulated in stimulated compared to unstimulated BMM. SDHA is the major catalytic subunit of the succinate-ubiquinone oxidoreductase. Altered SDHA expression may also have significant effects on oxidative phosphorylation (OXPHOS).

Short-term IFN-*α* stimulation of MDM was not associated with significant alterations in glycolytic genes or genes linked to lactate production (Figure 3, Supplementary Figure S3). Alternatively, stimulation was associated with the downregulation of genes associated with the conversion of galactose to glucose (*GALK2*, *GALT*, and *GALE*) and glycogen breakdown (*INSR*, *PHKA1*, *PHKA2*, *PHKG2*, and *AGL*). Early responses in MDM were associated with increased levels of phosphofructokinase (PFK) activator *PFKFB3*, which assists in the production of pyruvate from glucose and pyruvate dehydrogenase complex component X (*PDHX*), which may facilitate acetyl-CoA production from pyruvate. IFN-*α* was also associated with the upregulation of genes associated with OXPHOS including genes from complexes I and V of the electron transport chain (*NDUFA9*, *NDUFS4*, and *ATP5G3*) and the glycerol phosphate shuttle (glycerol 3-phosphate dehydrogenase 2 [*GPD2*]). Collectively, these results suggest that early changes in energy metabolism may play an important role in the initiation of antiviral responses in both BMM and MDM.

### 3.4. IFN-*α*-Stimulated BMM and MDM Show Signs of Alterations in Genes Associated with Redox Regulation

**Figure 4:**
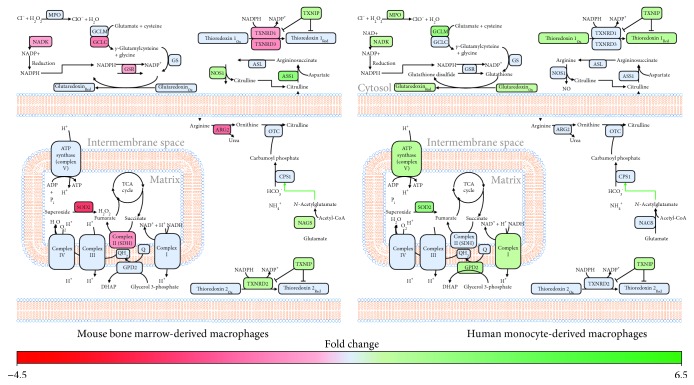
IFN-*α* stimulation of MDM is associated with increased expression of genes associated with ROS production and antioxidant responses. Differentially expressed metabolic genes (−1.2 ≤ FC ≥ 1.2, *p* value ≤ 0.05, FDR ≤ 0.1) were mapped to pathways associated with cellular redox status using MetScape and DAVID. Green and red represent genes that have been significantly upregulated or downregulated, respectively. Blue represents genes that were not altered following IFN-*α* stimulation.

Given the link between energy metabolism and ROS metabolism [45–47], we next examined alterations between early IFN-*α* responses and genes linked to cellular redox status (oxidant and antioxidant genes). In BMM, IFN-*α* short-term stimulation was associated with altered expression of genes associated with the nitric oxide cycle including the upregulation of argininosuccinate synthetase 1 (*ASS1*) and nitric oxide synthase 1 (*NOS1*), and downregulation of arginase 2 (*ARG2*) expression, which may favour flux of arginine towards NO production (Figure 4, Supplementary Figure S4). Early IFN-*α* responses in BMM were also associated with the downregulation of genes associated with the antioxidant response (superoxide dismutase 2 (*SOD2*), glutamate-cysteine ligase, catalytic subunit (*GCLC*), NAD kinase (*NADK*), and thioredoxin reductase 1 and 3 (*TXNRD1*, *TXNRD3*)) and the upregulation of thioredoxin interacting protein (*TXNIP*), which inhibits the antioxidant activity of thioredoxin [48, 49].

Alternatively, IFN-*α* stimulation of MDM was associated with the upregulation of antioxidant genes including SOD2 and myeloperoxidase (*MPO*) as well as genes associated with glutathione production (glutamate-cysteine ligase, modifier subunit (*GCLM*) and NAD^+^ kinase (*NADK*)) (Figure 4, Supplementary Figure S4). Short-term IFN-*α* was also associated with upregulation of and glutaredoxin (*GLRX*), thioredoxin 1 (*TXN1*), and thioredoxin-interacting protein (*TXNIP*). These alterations may help regulate electron linkage and subsequent ROS production associated with the upregulation of genes associated with OXPHOS in these cells (Figures 3 and 4).

### 3.5. Early Type I IFN Responses Are Associated with Alterations in Genes Associated with cAMP and cGMP Production

**Figure 5:**
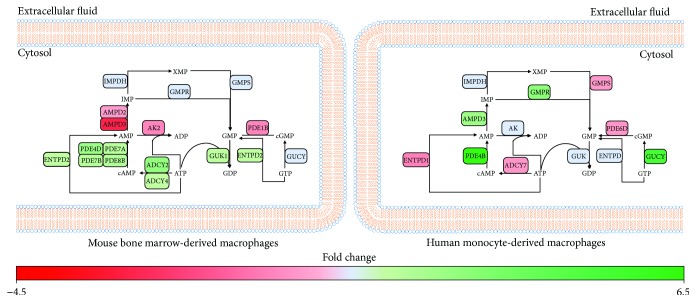
Type I IFN responses are associated with altered cAMP and cGMP production in BMM and MDM. Metabolic genes identified as significantly altered (−1.2 ≤ FC ≥ 1.2, *p* value ≤ 0.05, FDR ≤ 0.1) were mapped to pathways associated with AMP and GMP production using MetScape and DAVID. Green and red represent genes that have been significantly upregulated or downregulated, respectively. Blue represents genes that were not altered following IFN-*α* stimulation.

Given the enrichment of gene sets associated with nucleotide metabolism in both datasets, we examined the specific effects on short-term IFN-*α* stimulation on purine and pyrimidine metabolism. In BMM, short-term stimulation was associated with the downregulation of amidophosphoribosyltransferase (*PPAT*) and UMP synthetase (*UMPS*). These enzymes play a central role in ribose 5-phosphate incorporation during de novo purine and pyrimidine synthesis, which may represent an antiviral mechanism. At the level of purine degradation, IFN-*α* was associated with an upregulation of purine nucleoside phosphorylase (*PNP*), guanine deaminase (*GDA*), xanthine dehydrogenase (*XDH*), and ectonucleoside triphosphate diphosphohydrolase 2 and 5 (*ENTPD2*, *ENTPD5*) suggesting increased degradation. Interestingly, IFN responses were also associated with alterations in genes that regulate cyclic guanine monophosphate (cGMP)/GMP and cyclic adenosine monophosphate (cAMP)/AMP ratios (Figure 5, Supplementary Figure S5). Four phosphodiesterases (*PDE4D*, *PDE7A*, *PDE7B*, and *PDE8B*) and two adenylate cyclases (*ADCY2*, *ADCY4*) were upregulated, and adenylate kinase (*ADK*) and AMP deaminase 3 (*AMPD3*) were downregulated in stimulated versus unstimulated cells. These profiles suggest that IFN-*α*-activated BMM may accumulate both AMP and cAMP. IFN-*α* stimulation of BMM was also associated with the upregulation of guanylate kinase (*GUK*) and downregulation of phosphodiesterase 1B (*PDE1B*), suggesting these cells may favour cGMP production.

In MDM, IFN-*α* stimulation was not associated with significant changes in genes associated with de novo purine synthesis. However, IFN-*α* was associated with the downregulation of carbamoyl phosphate synthetase 2, aspartate transcarbamylase, and dihydroorotase (CAD), a protein responsible for the first three enzymatic steps of the pyrimidine biosynthesis pathway. Genes involved in nucleoside production within the purine and pyrimidine degradation pathways, such as GMP reductase (*GMPR*), *AMPD3*, 5′-nucleotidase, cytosolic II (*NT5C2*), and adenosine deaminase (*ADA*), are found to be upregulated in MDM which suggests an increase in nucleotide salvaging (Figure 5, Supplementary Figure S5). At the level of cGMP/GMP and cAMP/AMP regulation, the downregulation of *PDE6D* and adenylate cyclase 7 (*ADCY7*) as well as the upregulation of *PDE4B* and two soluble forms of guanylate cyclase (*GUCY1A3*, *GUCY1B3*) suggest a shift towards cGMP and AMP production. Varied expression of these bioactive nucleotides that function as intracellular secondary messengers may contribute to early type I IFN responses.

### 3.6. Short-Term IFN-*α* Stimulation Is Associated with Alterations in Tryptophan and Branched-Chain Amino Acid Catabolism in BMM and MDM

**Figure 6:**
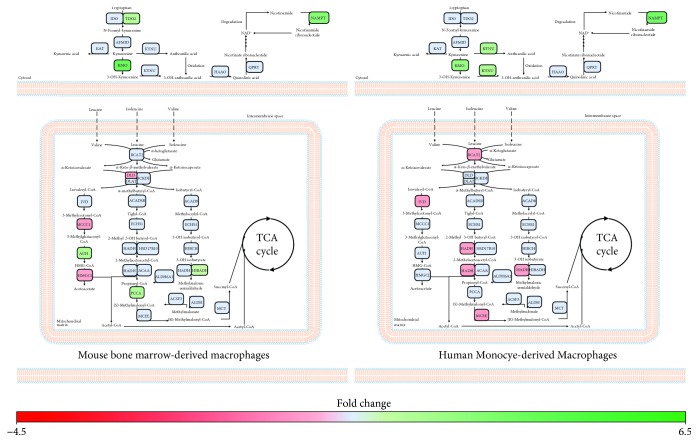
Tryptophan and branched-chain amino acid catabolism is altered in BMM and MDM following short-term IFN-*α* treatment. Metabolic genes altered in IFN-*α*-stimulated cells compared to controls (−1.2 ≤ FC ≥ 1.2, *p* value ≤ 0.05, FDR ≤ 0.1) were mapped to amino acid metabolism pathways using MetScape and DAVID. Green and red represent genes that have been significantly upregulated or downregulated, respectively. Blue represents genes that were not altered following IFN-*α* stimulation.

Consistent with the literature [50, 51], alterations in genes associated with tryptophan and branched-chain amino acid catabolism were pronounced in early IFN-*α* responses. Both BMMs and MDMs exhibited a pronounced upregulation of genes associated with tryptophan catabolism *via* the kynurenine pathway (Figure 6, Supplementary Figure S6). While IFN-*α*-stimulated BMM upregulated tryptophan 2,3-dioxygenase (*TDO2*) and *KMO*, stimulation of MDM was associated with the upregulation of *KMO* and kynureninase (*KYNU*). This shift in tryptophan catabolism was accompanied by the upregulation of nicotinamide phosphoribosyltransferase (*NAMPT*), suggesting an increased flux of tryptophan towards NAD^+^ production.

Both IFN-*α*-activated BMM and MDM showed altered expression of genes associated with branched-chain amino acid (Figure 6, Supplementary Figure S6). In BMMs, IFN-*α* stimulation leads to the upregulation of genes associated with isoleucine (propionyl-CoA carboxylase; *PPCA*, 3-ketoacyl-CoA thiolase 1A and 2; *ACAA1A*/*2*) and valine (3-hydroxyisobutyrate dehydrogenase; *HIBADH*) catabolism. Upregulation of AU RNA binding/methylglutaconyl-CoA hydratase (*AUH*) and downregulation of methylcrotonoyl-CoA carboxylase 1 (*MCCC1*) and 3-hydroxymethyl-3-methylglutaryl-CoA lyase (*HMGCL*) suggest decreased leucine catabolism in BMM. However, *AUH* may be functioning in its secondary role in promoting mRNA degradation [52]. In MDM, IFN-*α* stimulation was associated with a downregulation of multiple genes associated with branched-chain amino acid catabolism including branched-chain aminotransferase 2 (*BCAT2*), isovaleryl-CoA dehydrogenase (*IVD*), hydroxyacyl-CoA dehydrogenase (*HADH*), and methylmalonyl-CoA epimerase (*MCEE*). Together, this indicates that alterations in branched-chain amino acid catabolism may be key to driver of early IFN-*α* responses in primary macrophage systems.

### 3.7. IFN-*α* Stimulation Is Associated with Altered Lipid Metabolism in BMM and MDM

**Figure 7:**
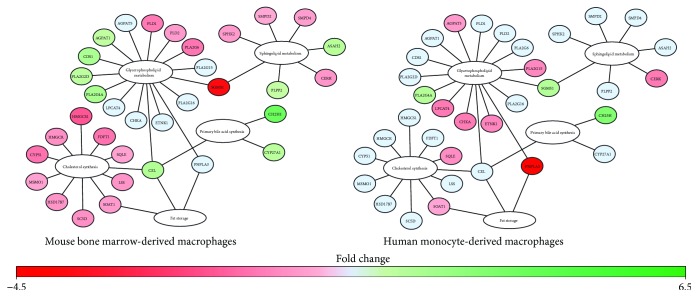
Expression of genes associated with lipid metabolism were differentially modulated in IFN-*α*-stimulated BMM compared MDM. Differentially expressed metabolic genes (−1.2 ≤ FC ≥ 1.2, *p* value ≤ 0.05, FDR ≤ 0.1) involved in cholesterol metabolism and phospholipid and sphingolipid synthesis were mapped using MetScape and DAVID. Green and red represent genes that have been significantly upregulated or downregulated, respectively. Blue represents genes that were not altered following IFN-*α* stimulation.

Lipid metabolism has been shown to play an important role in antiviral responses in BMM and MDM [53]. Several studies have reported alterations in cholesterol metabolism during IFN and antiviral responses [21–23]. Here, we also identified alterations in genes associated with phospholipid and sphingolipid metabolism and FA biosynthesis following short-term IFN responses. Consistent with previous studies, short-term IFN-*α* stimulation of BMM and MDM was associated with the downregulation of genes associated with de novo cholesterol synthesis (Figure 7, Supplementary Figure S7). In BMM, IFN-*α* was associated with the downregulation of genes involved in mevalonate synthesis (*HMGCS1*, *HMGCR*), lanosterol synthesis (*FDFT1*, *SQLE*, and *LSS*) and cholesterol synthesis (*CYP51*, *MSMO1*, *HSD17B7*, and *SC5D*). It was also associated with the downregulation of genes associated with cholesterol ester formation and the upregulation of carboxyl ester lipase (*CEL*), cholesterol 25-hydroxylase (*CH25H*), and sterol 27-hydroxylase (*CYP27A1*). In MDM, IFN-*α* stimulation was associated with decreased levels of *SQLE* and sterol O-acyltransferase 1 (*SOAT1*) and increased levels of *CH25H*. SQLE catalyzes the first oxygenation step in sterol biosynthesis and is thought to be a rate-limiting enzyme of this process [54].

At the level of phospholipid and sphingolipid metabolism, IFN-*α*-treated BMM upregulated phospholipid phosphatase 2 (*PLPP2*) and neutral ceramidase (*ASAH2*) and downregulated sphingolipid kinases (*SPHK2*, *CERK*) suggesting a shift away from phosphorylated sphingolipids to sphingosine in acute IFN responses (Figure 7, Supplementary Figure S7). Stimulation of BMM was also associated with the upregulation of 1-acylglycerol-3-phosphate O-acyltransferase 1 (*AGPAT1*) and phosphatidate cytidylyltransferase 1 (*CDS1*), which may increase cytidine diphosphate- (CDP-) diacylglycerol production, a precursor for phosphatidylinositol, phosphatidylglycerol, and cardiolipin synthesis. Phosphatidylinositol is a minor component on the cytosolic side of cell membranes, and cardiolipin is an important component of the inner mitochondrial membrane [55]. Consistent with these findings, IFN-*α* stimulation was associated with the upregulation of three different phospholipase A2 (*PLA2G2D*, *PLA2G4A*, and *PLA2G16*) genes and the downregulation of two phospholipase D (*PLD1*, *PLD2*) genes. These genes cleave phosphatidylcholine and phosphatidylethanolamine, which represent the major phospholipids in mammalian membranes. The relative ratio of these lipids to one another within the cell membrane has significant implications on membrane integrity [55]. In MDM, *PLA2G4A* and sphingomyelin synthase 1 (*SGMS1*) were upregulated and lysophospholipid acyltransferase (*LPCAT4*) and *PLA2G15* were downregulated following IFN-*α* stimulation (Figure 7, Supplementary Figure S7). IFN-*α* stimulation was also associated with the downregulation of *AGPAT5*, choline kinase alpha (*CHKA*), and ethanolamine kinase 1 (*ETNK1*), which play an important role in the synthesis of phosphatidylglycerol, phosphatidylcholine, and phosphatidylethanolamine. Collectively, these alterations suggest IFN-*α* responses may alter the composition of the plasma and mitochondrial membranes of BMM and MDM as part of early type I IFN responses.

Finally, at the level of fatty acid synthesis, IFN-*α* stimulation of BMM was also associated with the downregulation of FA synthase (*FAS*) and FA desaturase 1 (*FADS1*) as well as the upregulation of carnitine palmitoyltransferase 1A (*CPT1A*). Alternatively, in MDM, IFN-*α* was associated with the upregulation of long-chain fatty acid- (LCFA-) producing aldehyde dehydrogenase 3b1 (*ALDH3B1*) and three acyl-CoA synthetase long-chain genes (*ACSL1*, *ACSL5*, and *ACSL6*) and the downregulation of carbonyl reductase 4 (*CBR4*), acetyl-CoA carboxylase-*α* (*ACACA*), mitochondrial 3-oxoacyl-ACP synthase (*OXSM*), trimethyllysine hydrolase *ε* (*TMLHE*), and hydroxyacyl-CoA dehydrogenase (*HADH*). Differences in fatty acid metabolism may indicate differential dependencies of BMM and MDM on *β*-oxidation for energy production.

## 4. Discussion

In the current study, we used publicly available transcriptional profiling datasets to develop metabolic gene signatures associated with short-term IFN-*α* stimulation in mouse and human macrophage models. Enrichment analysis, pathway mapping, and network construction identified alterations in central metabolic pathways in early IFN-*α* responses including glycolysis, oxidative phosphorylation, redox regulation, nucleotide metabolism, amino acid catabolism, and lipid metabolism. BMM had increased expression of genes associated with aerobic glycolysis, nitric oxide production, branched-chain amino acid metabolism, and fatty acid *β*-oxidation as well as decreased expression of genes associated with cholesterol biosynthesis. MDM had increased expression of genes associated with increased OXPHOS activity and antioxidant production and decreased expression of genes associated with branched-chain amino acid catabolism and fatty acid *β*-oxidation. While the current study only examines alterations in gene expression, these findings suggest that metabolic rewiring, at the level of transcription, is a key feature of early IFN-*α* responses. Future studies are required to validate the identified gene signatures and to validate the biological relevance of these alterations during early antiviral immune responses.

A number of studies have reported increased aerobic glycolysis and reduced oxidative phosphorylation in macrophages following activation with inflammatory stimuli [56–59]. Consistent with the literature, short-term IFN-*α* stimulation of BMM was associated with increased expression of genes associated with glycolysis (*HK2*, *HK3*, *PGM2*, *PFKP*, *PFKFB3*, and *INSR*) and lactate production (*LDHD*) and decreased expression of genes associated with pyruvate production (*PDK3*, *PDP1*, and *DLD*) and flux through the TCA cycle (*DLD*, *DLST*, and *SDHA*). In addition to meeting bioenergetic requirements of the cells, these alterations may increase intracellular levels of bioactive metabolites such as d-lactate, *α*-ketoglutarate, and succinate. Lactate accumulation in the microenvironment has been shown to suppress cytokine production and migration of human cytotoxic T cells [60, 61]. Similarly, *α*-ketoglutarate has been shown to quell inflammatory processes by suppressing NF-*κ*B-mediated inflammatory pathways [62]. Succinate can also modulate inflammatory cytokine production. A recent study found that succinate stabilizes HIF-1*α* expression in LPS-activated macrophages [14], which facilitates HIF-1*α* transport into the nucleus where it induces the expression of glycolytic targets such as *LDHA*, *HK2*, and *PKM2*, as well as inflammatory genes such as *IL1B* [63, 64]. Inhibition or decreased expression of succinate dehydrogenase (SDH) has been shown to promote IL-10 expression and to repurpose the mitochondria for ROS production [19]. While functional studies are required to validate these profiles, our findings suggest bioenergetic reprogramming of BMM may represent a feedforward mechanism that may contribute to the immunomodulatory properties of type I IFNs during early antiviral immune responses.

Unlike BMM, short-term IFN-*α* stimulation of MDM was associated with the upregulation of a range of genes associated with the electron transport chain, which may favour OXPHOS for energy production. While OXPHOS provides more ATP per glucose molecule compared to aerobic glycolysis, energy and metabolic precursor production occurs more slowly. Further, IFN-*α* stimulation of MDM was associated with the downregulation of genes associated with the Leloir pathway (*GALK2*, *GALT*, and *GALE*), which is responsible for the conversion of galactose to glucose. Recent studies have shown that T cells, but not B cells, can be activated and proliferate in the presence of galactose when glucose is absent [65, 66]. However, unlike activation in glucose-rich environments, T cells in galactose are forced to rely on OXPHOS for energy production, which occurs at significantly slower rates [65]. This reliance on galactose also results in suboptimal IFN-*γ* and IL-2 production suggesting galactose should only be used when no other energy substrate is available [65]. Thus, decreased expression of genes associated with the Leloir pathway in MDM may represent a means by which cells can improve the efficiency of energy production while maintaining functional immune responses.

Transcriptional profiling also identified redox regulation as a key feature of early IFN-*α* responses in mouse and human primary macrophage models. In BMM, IFN-*α* reprogramming was associated with increased expression of genes associated with nitric oxide (NO) production (*ASS1*, *NOS1*, and *NAGS*) and decreased antioxidants (*SOD2*, *GCLC*, *TXNRD1*, and *TXNRD3*). NO is a potent antimicrobial molecule that has been shown to modulate cellular metabolism [67–70] and immune function [71]. *ASS1* and *ASL* are part of the aspartate-argininosuccinate shunt, which recycles citrulline to resynthesize arginine for prolonged NO production [72, 73]. In M1 macrophages, increased expression of ASS1 and the subsequent increased flux through this shunt has been shown to replenish TCA cycle intermediates following decreased *IDH* and *SDH* gene expression [72]. Unlike BMM, IFN-*α*-stimulated MDM had increased expression of genes associated with OXPHOS and ROS production. ROS are also potent antimicrobial molecules, capable of killing intracellular pathogens [74]. The matched upregulation of antioxidant genes (e.g., glutathione, glutaredoxin, and thioredoxin) in conjunction with ROS likely reflects a protective mechanism to limit any associated cellular damage. Interestingly, a recent study found that the reducing nature of glutathione can prime T cell inflammatory responses by promoting mTOR-activated metabolic reprogramming [75]. It is currently unclear if similar priming occurs in macrophages. Collectively, our data suggest mouse BMM and human MDM may adopt differential metabolic strategies to mount intracellular antimicrobial responses during acute IFN responses.

Pathway mapping and network reconstruction identified IFN-*α*-associated alterations in nucleotide metabolism. In both BMM and MDM, IFN-*α* stimulation was associated with a downregulation of genes associated with de novo pyrimidine biosynthesis. A number of viruses including human cytomegalovirus and herpes simplex viruses require de novo pyrimidine synthesis for propagation and survival [76, 77]. Furthermore, inhibitors of de novo pyrimidine biosynthesis have broad antiviral effects against RNA, DNA, and retroviruses such as influenza A, hepatitis C, human adenovirus, and human immunodeficiency virus (HIV) [78, 79]. In MDM, IFN-*α* stimulation was also associated with increased expression of genes associated with purine and pyrimidine degradation pathways and nucleotide salvaging. The induction of nucleotide degradation pathways may act as a counterstrategy against viral-driven nucleotide biosynthesis [80]. Moreover, the activation of nucleotide salvaging pathways may allow the cell to recycle degraded bases and nucleosides and produce nucleotides to maintain cellular function. Interestingly, IFN-*α* responses were also associated with alterations in genes that regulate cGMP/GMP and cAMP/AMP ratios. Cyclic nucleotide second messengers, including cAMP and cGMP, are potent secondary messengers that contribute to the regulation of a variety of cellular processes including metabolism [81]. cAMP has been shown to suppress innate immune function including inflammatory cytokine production, cell adhesion, phagocytosis, and intracellular killing [82, 83]. Additionally, the cAMP axis plays an important role in antimicrobial defense as many microbes have evolved virulence-enhancing strategies that exploit this pathway [84–86]. In BMM, LPS responses are associated with low levels of cAMP and cGMP accumulation, which inhibit inflammatory cytokine production [87, 88]. Thus, alterations in the cAMP/AMP and cGMP/GMP ratio may play an important role in regulating inflammatory, antimicrobial, and metabolism responses in acute type I IFN responses.

Alterations in genes associated with tryptophan and branched-chain amino acid catabolism were pronounced in early IFN responses. Consistent with previous studies [50, 89, 90], IFN-*α* stimulation was associated with increased levels of genes associated with tryptophan catabolism. While we did not observe alterations in *IDO1* expression, *TDO2*, *KMO*, and *KYNU* were increased in both BMM and MDM. Many studies have shown that tryptophan catabolism (via IDO activation) represents a potent antiviral immune response [90–92]. Our study suggests downstream enzymes of the kynurenine pathway may also contribute to this phenotype. Interestingly, we also found that *NAMPT* was upregulated following acute IFN-*α* stimulation, suggesting tryptophan may be directed towards NAD^+^ salvaging. NAMPT plays an important role in regulating glycolytic flux, phagocytic activity, and TNF-*α* production in LPS-stimulated macrophages and may also contribute to IFN responses [93, 94]. At the level of branched-chain amino acid metabolism, BMM had increased expression of genes associated with branched-chain amino acid catabolism. Conversely, MDM downregulated genes associated with this pathway. Catabolic products of branched-chain amino acid metabolism feed into the TCA cycle contributing to the production of succinyl-CoA and acetyl-CoA [95]. Previous studies have shown that branched-chain amino acid availability is critical for lymphocyte proliferation and M1 macrophage activation, but little is known regarding the role of these amino acids in regulating immune responses [96, 97]. Thus, upregulation of branched-chain amino acid catabolism in BMM may compensate for the loss of OXPHOS activity, which is not required in MDM.

Lipid metabolism has been shown to play an important role in antiviral responses in BMM and MDM [23, 24, 53]. Consistent with the literature, altered gene expression in BMM and MDM suggest these cells may downregulate de novo cholesterol synthesis and shunt available free cholesterol towards the production of oxysterols including 25-hydroxycholesterol and 27-hydroxycholesterol. The role of cholesterol flux in antiviral responses has been described previously [21–23]. Increased cholesterol levels help facilitate the entry of the dengue virus during the early phases of infection, which is reduced by the presence of oxysterols such as 25-hydroxycholesterol [22, 98–100]. Macrophages can also counteract this demand by switching away from de novo synthesis towards lipid import [23]. Limiting flux through the cholesterol biosynthetic pathway induces a STING-mediated type I IFN response, which can be attenuated by exogenous free cholesterol [21, 23]. We also observed significant alterations in genes associated with phospholipid, sphingolipid, and FA metabolism. Lipid membrane composition can play a critical role in the antiviral capabilities of immune cells. Sphingolipids and phosphatidylserine have been shown to function as receptors for polyomavirus, HIV, and vesicular stomatitis virus (VSV) [101–103]. Altering the lipid composition of the plasma membrane is a vital protective strategy against viral entry by altering the potential interaction sites for viruses [104, 105]. Thus, altering the membrane lipid composition may be a critical feature of metabolic reprogramming in early antiviral responses.

Collectively, our study provides critical new insights into the molecular underpinnings of metabolic reprogramming associated with short-term IFN-*α* responses in mouse BMM and human MDM. This is the first study to systematically characterize changes in metabolic gene expression using transcriptional profiling in this context. However, we acknowledge certain limitations of this study. The current study only evaluates gene expression profiles via microarray. Validation and functional testing is required to understand the biological relevance of these findings. While both BMM and MDM were stimulated with short-term IFN-*α*, we cannot exclude the possibility that some of the reported differences may reflect the length of time in stimulant (2.5 h versus 4 h). Preliminary studies from our laboratory suggest metabolic profiles in stimulated BMM are similar within a 2–6-hour window. However, future time-course studies are required to examine how metabolic signatures change in BMM and MDM over short- and long-term IFN-*α* stimulation. Culture conditions and differentiation protocols may also affect metabolic profiles in BMM versus MDM. This limitation should be an important consideration across all studies examining relationships between immune and metabolic processes in vitro, whether in humans or in mice. Careful consideration of the model system may be required depending on downstream applications of the findings. Meta-analyses of transcriptional datasets may represent a powerful tool to identify metabolic signatures that are consistently altered across different studies using different models, time points, culture conditions, and so on. To minimize these effects, only studies performed in high glucose DMEM (plus glutamine and sodium pyruvate) with 10% FBS were selected for analyses. Finally, both BMM and MDM were analyzed using Affymetrix technologies' microarray chips. However, we cannot exclude the possibility that the reported differences may be affected by the microarray used. Despite these limitations, we strongly believe this comparative study provides important new insights into metabolic processes that contribute to IFN responses in mouse and human macrophages. We believe that the power of an untargeted approach such as transcriptional profiling is to systematically characterize these differences, which may have important implications on effector function depending on the local microenvironment. In the future, more targeted studies are required to evaluate the effects of these gene expression profiles on protein expression and functional metabolic and immune responses.

## 5. Conclusions

In summary, this study identified a variety of metabolic pathways altered following short-term IFN-*α* stimulation in mouse and human macrophage systems. This may have important implications for the initiation of early antiviral immune responses, including the induction of the specific antimicrobial and immunomodulatory functions of IFN-*α*. While functional studies are required to clearly elucidate the relationships between this metabolic reprogramming and effector function, it is clear that transcriptional regulation of metabolic processes is a key feature of early type I IFN responses. An in-depth understanding of this early reprogramming may lead to the development of targeted therapeutics that regulate and fine tune specific type I IFN effector function.

## Data Availability

Gene expression data used in this study have been previously published in the GEO database under the accession numbers GSE16755 (https://www.ncbi.nlm.nih.gov/geo/query/acc.cgi?acc=GSE16755) and GSE35825 (https://www.ncbi.nlm.nih.gov/geo/query/acc.cgi?acc=GSE35825).

## Supplementary Materials

Supplementary Table S1 A list of all metabolic genes identified in the BMM dataset.

Supplementary Table S2 A list of all metabolic genes identified in the MDM dataset.

Supplementary Table S3 A list of significantly altered gene sets identified by GSEA in the BMM dataset.

Supplementary Table S4 A list of significantly altered gene sets identified by GSEA in the MDM dataset.

Supplementary Figure S1 Workflow of BMM and MDM differentiation and IFN-*α* stimulation. Mouse bone marrow cells and human monocytes were differentiated for 7 days into BMM and MDM, respectively. Cells were then stimulated with IFN-*α* for less than 4 hours. RNA was extracted from total cells and prepared for microarray analysis.

Supplementary Figure S2 IFN-*α* is associated with differential enrichment of metabolic pathways in mouse BMM compared to human MDM. Metabolite set enrichment analysis (MSEA) was performed in MetaboAnalyst using metabolic gene datasets. Pathways shown were significantly enriched (*P* < 0.05) in either IFN-stimulated BMM, MDM, or both. Yellow and blue represent enrichment scores in BMM and MDM, respectively (error bars = sem; *n* = 3).

Supplementary Figure S3 IFN-*α* responses are associated with altered bioenergetic profiles in mouse and human macrophages. The bar plots show significantly altered genes associated with bioenergetics pathways (FC > 1.2, *p* < 0.05, FDR < 0.10). Light blue and dark blue represent expression levels in unstimulated (control) and IFN-*α*-treated macrophages, respectively (error bars = sem; *n* = 3).

Supplementary Figure S4 BMM and MDM express redox-related genes following short-term IFN-*α* stimulation. The bar plots show significantly altered genes associated with cellular redox pathways (FC > 1.2, *p* < 0.05, FDR < 0.10). Light blue and dark blue represent expression levels in unstimulated (control) and IFN-*α*-treated macrophages, respectively (error bars = sem; *n* = 3).

Supplementary Figure S5 IFN-*α* is associated with altered expression of genes that regulate nucleotide metabolism and cAMP/cGMP ratios. The bar plots show significantly altered genes associated with nucleotide metabolism (FC > 1.2, *p* < 0.05, FDR < 0.10). Light blue and dark blue represent expression levels in unstimulated (control) and IFN-*α*-treated macrophages, respectively (error bars = sem; *n* = 3).

Supplementary Figure S6 Short-term IFN-*α* stimulation is associated with alterations in tryptophan and branched chain amino acid catabolism. The bar plots show significantly altered genes associated with tryptophan and branched chain amino acid metabolism (FC > 1.2, *p* < 0.05, FDR < 0.10). Light blue and dark blue represent expression levels in unstimulated (control) and IFN-*α*-treated macrophages, respectively (error bars = sem; *n* = 3).

Supplementary Figure S7 Altered expression of genes associated with lipid metabolism is a key feature of IFN-*α* responses. The bar plots show significantly altered genes associated with lipid metabolism (FC > 1.2, *p* < 0.05, FDR < 0.10). Light blue and dark blue represent expression levels in unstimulated (control) and IFN-*α*-treated macrophages, respectively (error bars = sem; *n* = 3).
