# Supplementary figures and images for: Transcriptional Profiling Suggests Extensive Metabolic Rewiring of Human and Mouse Macrophages during Early Interferon Alpha Responses
Source: Mediators Inflamm. 2018 Jul 25;2018:5906819. doi: 10.1155/2018/5906819 (PMC6083555; doi:10.1155/2018/5906819)

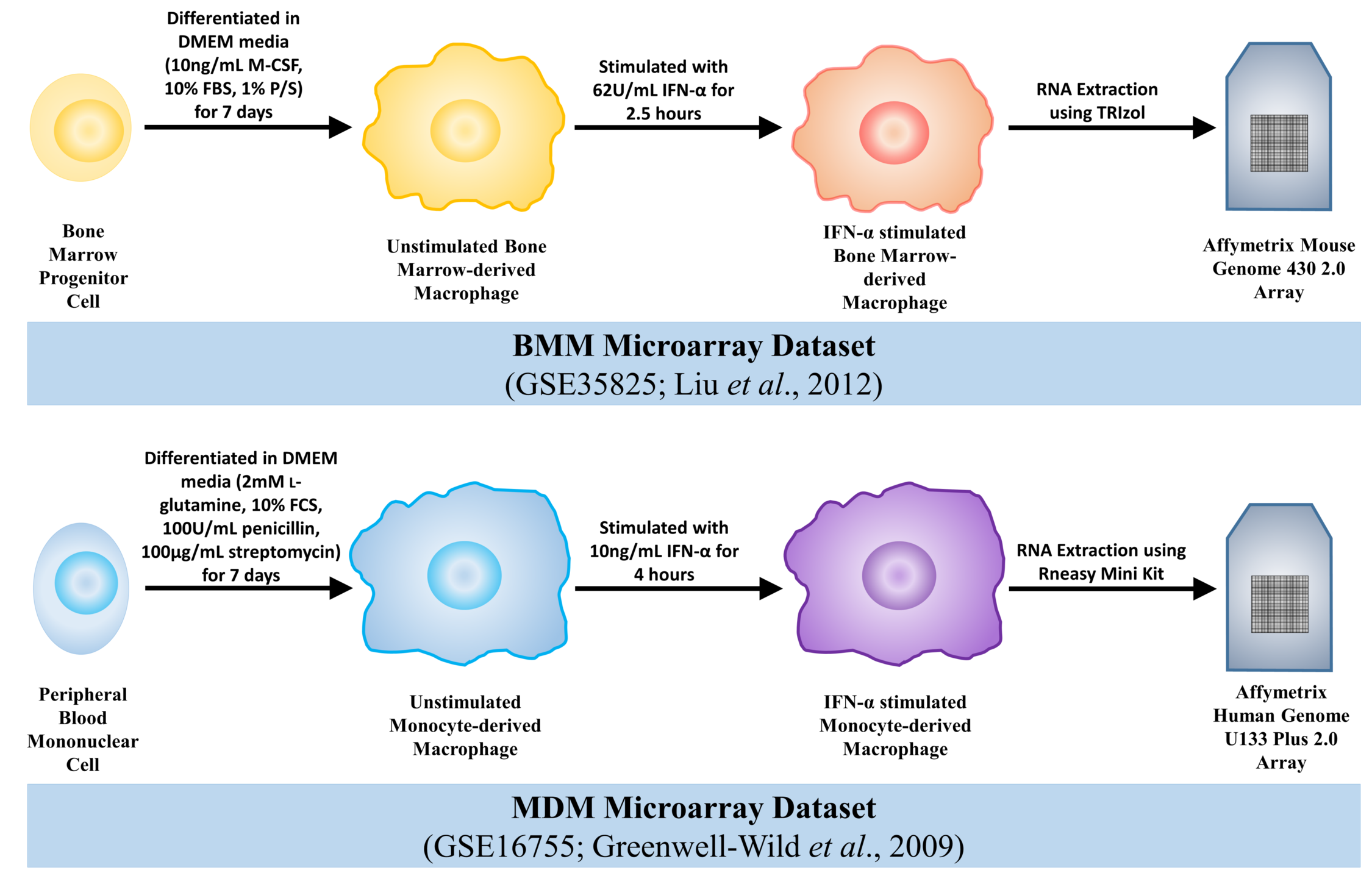

Supplement: Supplementary Figure S1 — Workflow of BMM and MDM differentiation and IFN-α stimulation. Mouse bone marrow cells and human monocytes were differentiated for 7 days into BMM and MDM, respectively. Cells were then stimulated with IFN-α for less than 4 hours. RNA was extracted from total cells and prepared for microarray analysis. [file 5906819.f5.tiff]

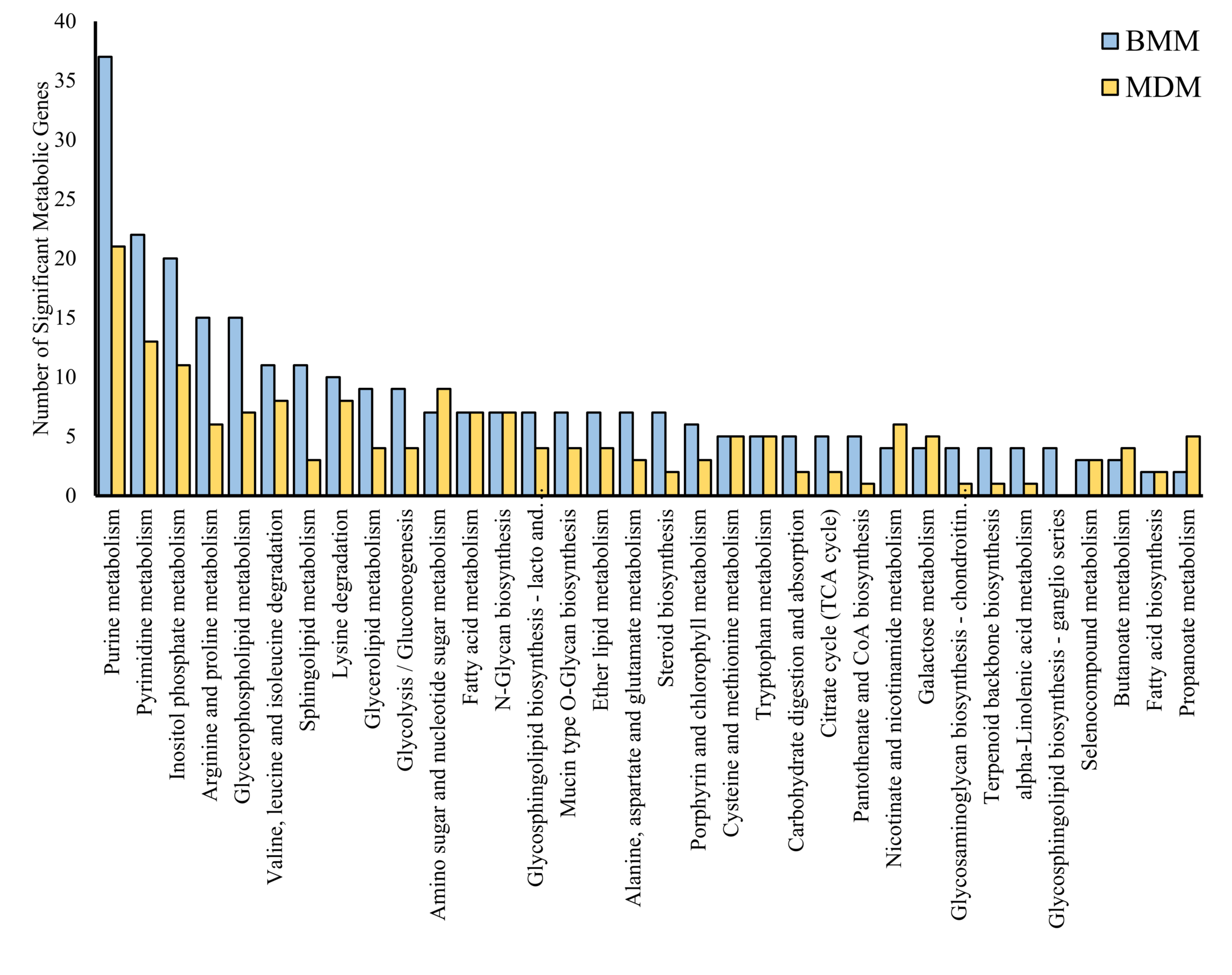

Supplement: Supplementary Figure S2 — IFN-α is associated with differential enrichment of metabolic pathways in mouse BMM compared to human MDM. Metabolite set enrichment analysis (MSEA) was performed in MetaboAnalyst using metabolic gene datasets. Pathways shown were significantly enriched (P < 0.05) in either IFN-stimulated BMM, MDM, or both. Yellow and blue represent enrichment scores in BMM and MDM, respectively (error bars = sem; n = 3). [file 5906819.f6.tiff]

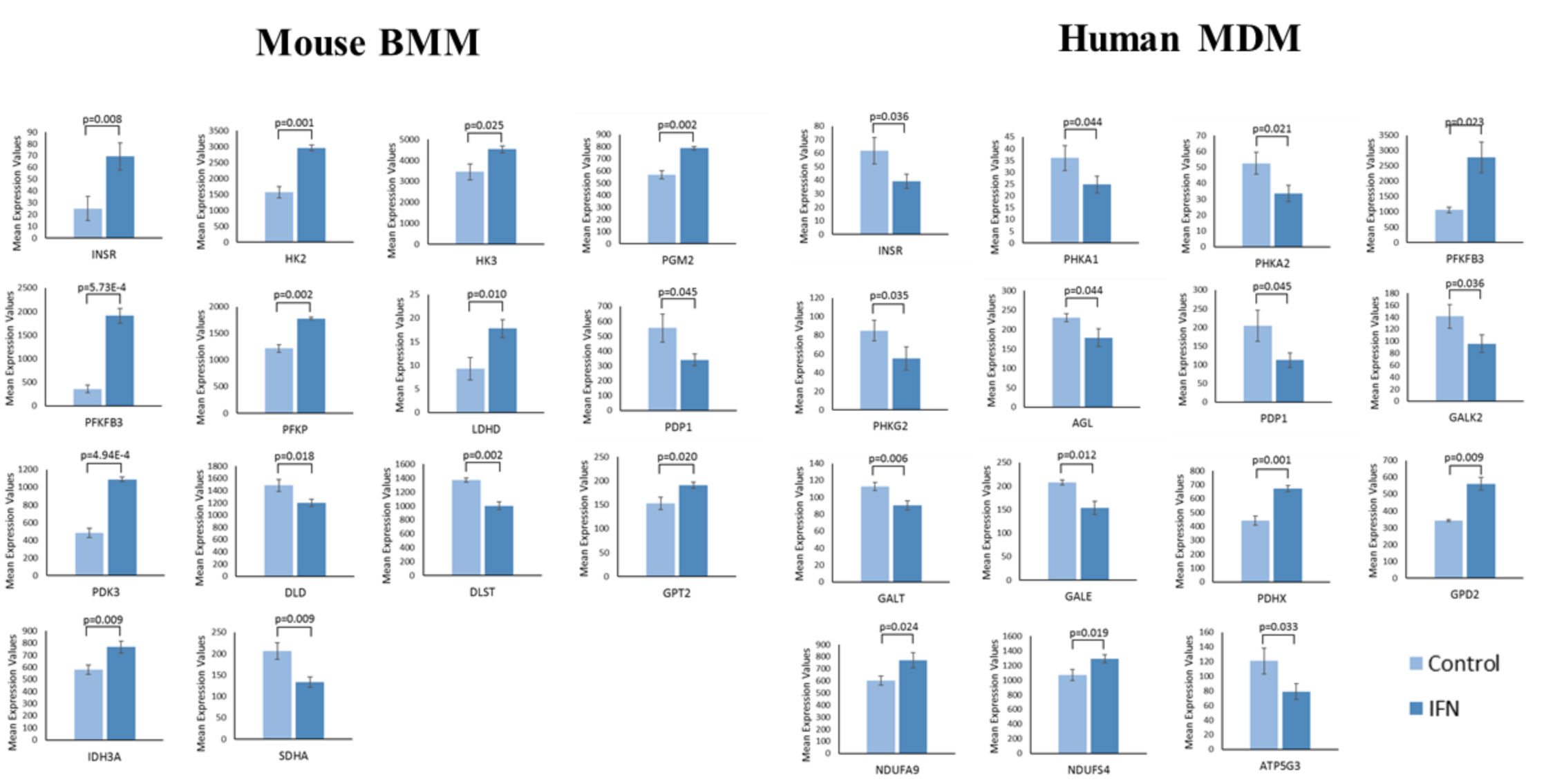

Supplement: Supplementary Figure S3 — IFN-α responses are associated with altered bioenergetic profiles in mouse and human macrophages. The bar plots show significantly altered genes associated with bioenergetics pathways (FC > 1.2, p < 0.05, FDR < 0.10). Light blue and dark blue represent expression levels in unstimulated (control) and IFN-α-treated macrophages, respectively (error bars = sem; n = 3). [file 5906819.f7.tiff]

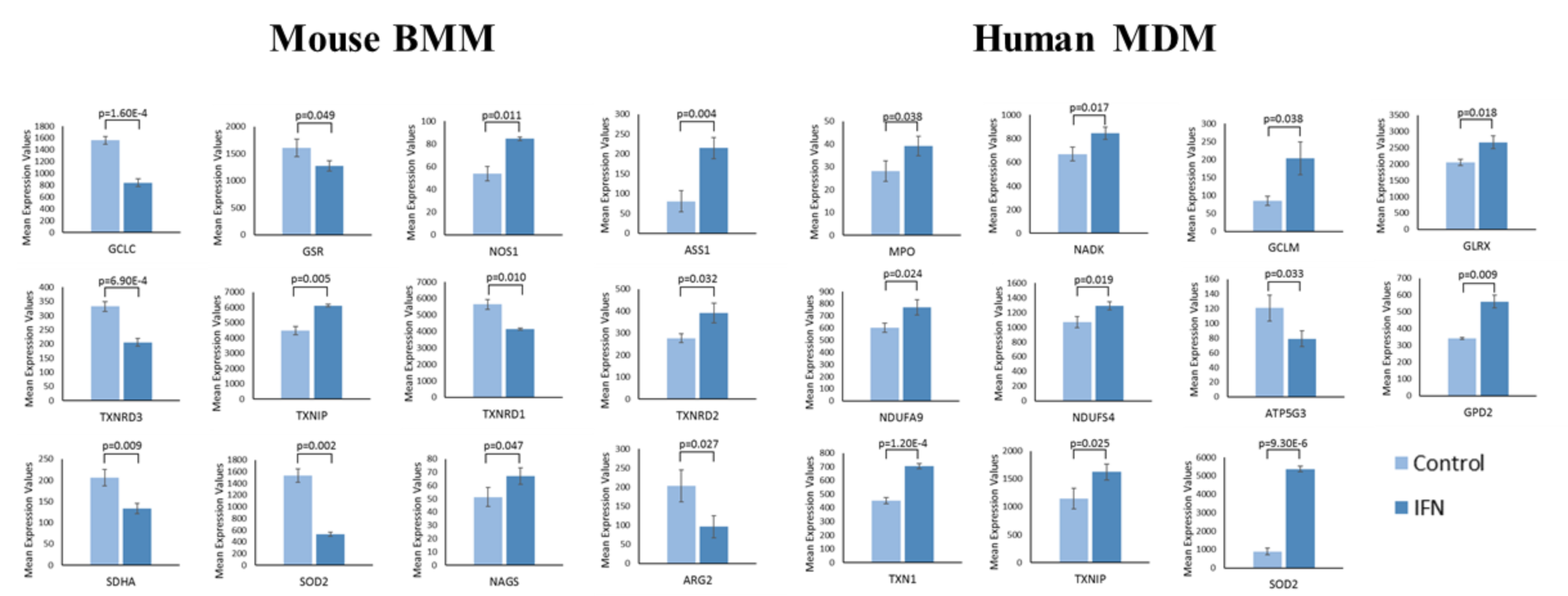

Supplement: Supplementary Figure S4 — BMM and MDM express redox-related genes following short-term IFN-α stimulation. The bar plots show significantly altered genes associated with cellular redox pathways (FC > 1.2, p < 0.05, FDR < 0.10). Light blue and dark blue represent expression levels in unstimulated (control) and IFN-α-treated macrophages, respectively (error bars = sem; n = 3). [file 5906819.f8.tiff]

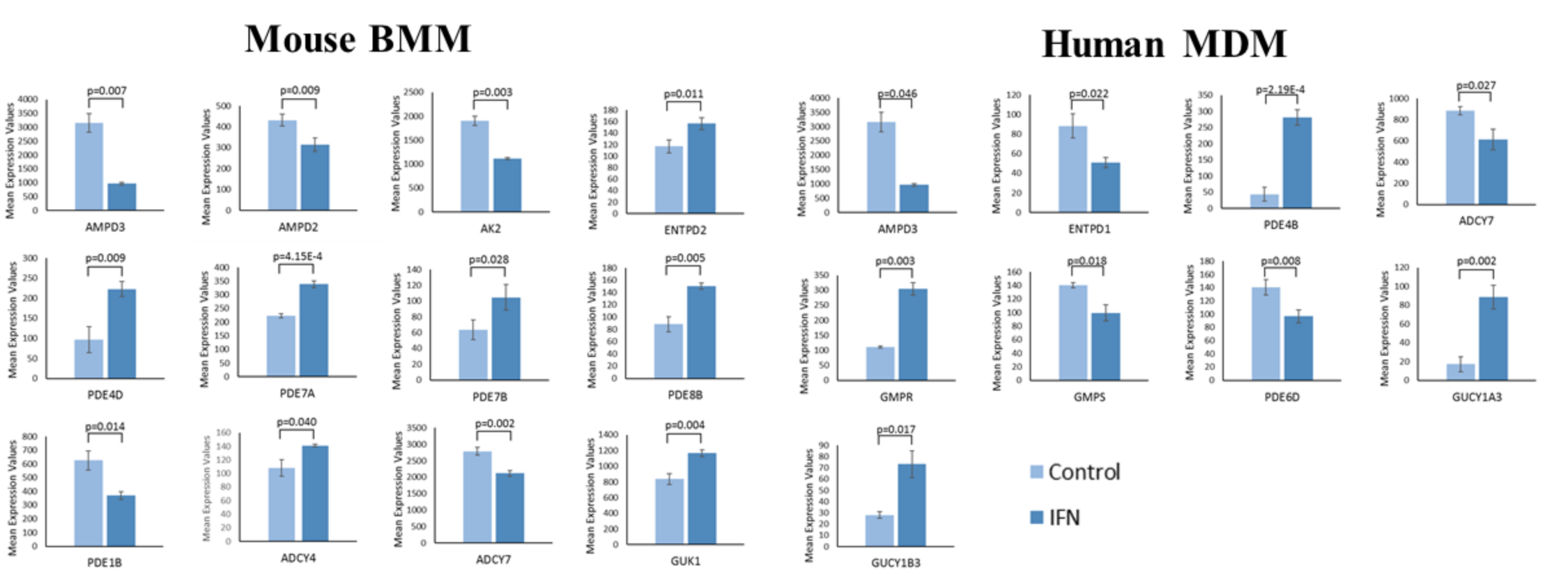

Supplement: Supplementary Figure S5 — IFN-α is associated with altered expression of genes that regulate nucleotide metabolism and cAMP/cGMP ratios. The bar plots show significantly altered genes associated with nucleotide metabolism (FC > 1.2, p < 0.05, FDR < 0.10). Light blue and dark blue represent expression levels in unstimulated (control) and IFN-α-treated macrophages, respectively (error bars = sem; n = 3). [file 5906819.f9.tiff]

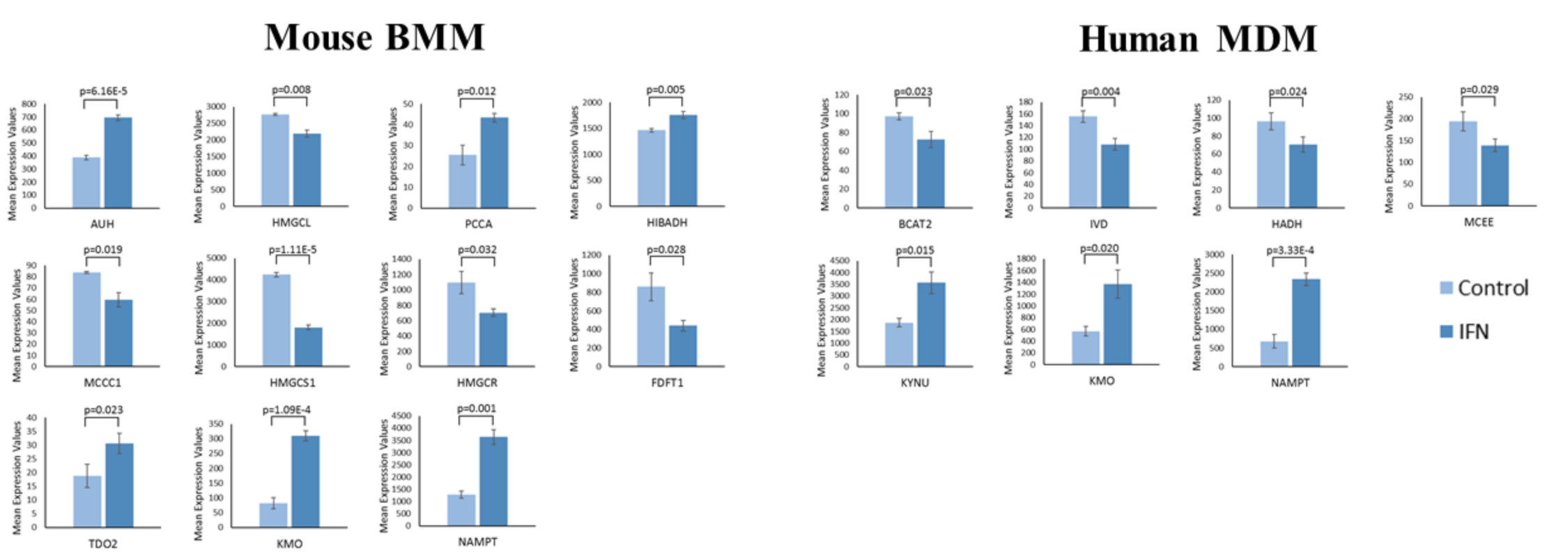

Supplement: Supplementary Figure S6 — Short-term IFN-α stimulation is associated with alterations in tryptophan and branched chain amino acid catabolism. The bar plots show significantly altered genes associated with tryptophan and branched chain amino acid metabolism (FC > 1.2, p < 0.05, FDR < 0.10). Light blue and dark blue represent expression levels in unstimulated (control) and IFN-α-treated macrophages, respectively (error bars = sem; n = 3). [file 5906819.f10.tiff]

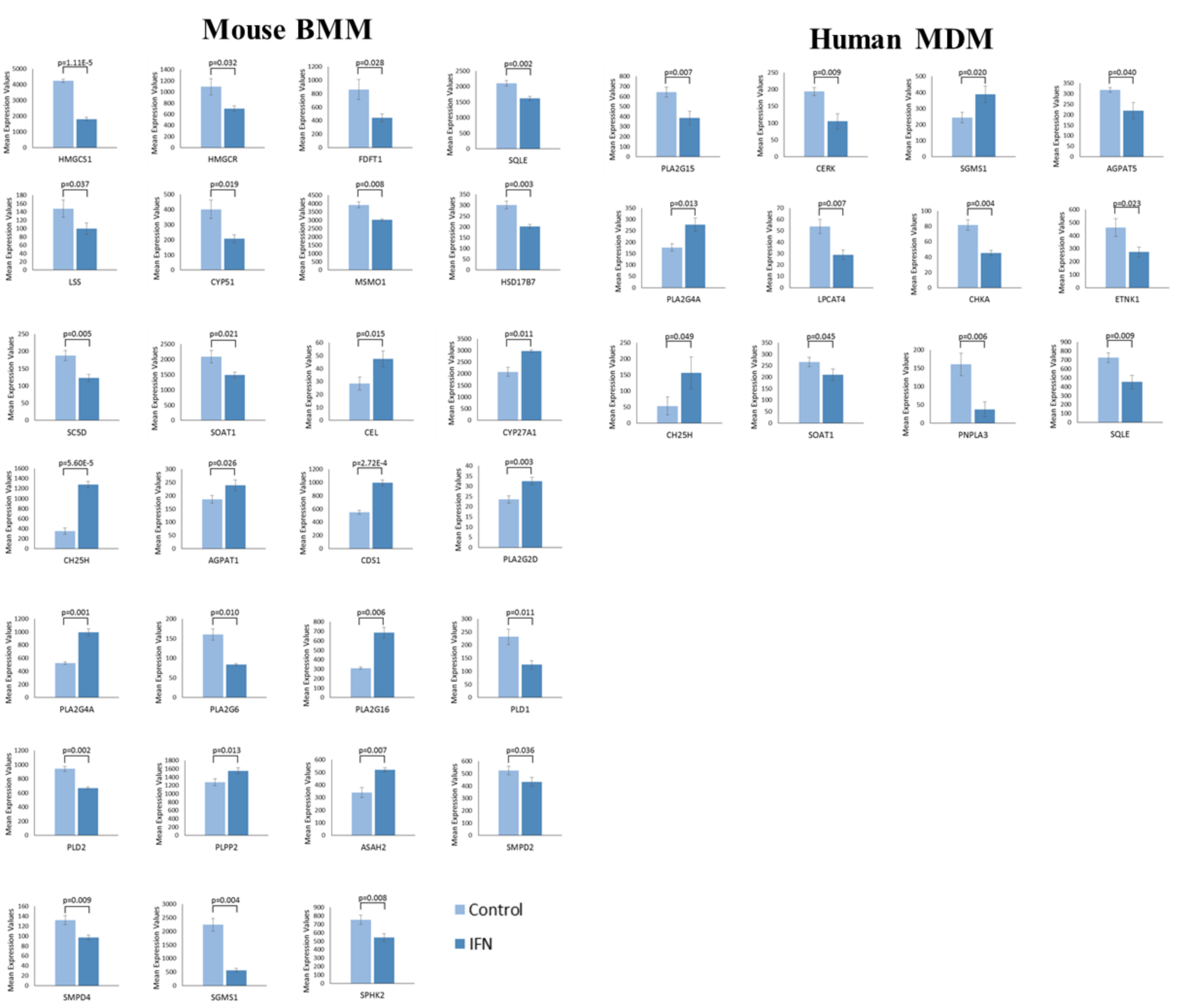

Supplement: Supplementary Figure S7 — Altered expression of genes associated with lipid metabolism is a key feature of IFN-α responses. The bar plots show significantly altered genes associated with lipid metabolism (FC > 1.2, p < 0.05, FDR < 0.10). Light blue and dark blue represent expression levels in unstimulated (control) and IFN-α-treated macrophages, respectively (error bars = sem; n = 3). [file 5906819.f11.tiff]
